# Escalating Fire‐Driven Forest Loss in Protected Tropical Forests

**DOI:** 10.1111/gcb.71051

**Published:** 2026-08-11

**Authors:** Christopher G. Bousfield, Oscar Morton

**Affiliations:** ^1^ Ecology and Evolutionary Biology, School of Biosciences The University of Sheffield Sheffield UK

**Keywords:** biodiversity, climate change, conservation, fire, protected area, tropical forest

## Abstract

Wildfires are driving substantial losses of tropical forest, with climate change expected to increase fire‐driven losses throughout the century. Whether Protected Areas (PAs)—and the vital habitat they protect—are buffered from fire is a key question. Here we combine PA and stand‐replacing fire data to estimate that > 4 million hectares (Mha) of protected tropical forest have been lost to stand‐replacing fire since 2001, with one‐third of losses (1.35 Mha) occurring in 2024 alone. The vast majority of losses (89%) were concentrated in the tropical Americas (3.6 Mha), most of which occurred in Brazil (2.7 Mha), followed by Asia‐Pacific (0.31 Mha) and Africa (0.14 Mha). Increasing trends in annual losses were observed across the Americas and Africa, occurring in 30 countries across the tropics. Counterfactual analysis suggests that despite considerable heterogeneity PAs most often reduce the probability of stand‐replacing fires relative to non‐PAs across the tropics. In the Americas, protection reduced stand‐replacing fires in 48% of protected plots, rising to 71% and 55% in Africa and Asia‐Pacific, respectively. However, temporal burn trends remained tightly coupled inside and outside of PAs, suggesting PAs are equally exposed to increasing global trends in fire activity. Ensuring existing PAs are protected from anthropogenic ignition sources is a priority, but subsequent PA planning must consider future fire risk to ensure they remain an effective conservation tool in a changing climate.

## Graphical abstract

More than 4 million hectares of protected tropical forest have been lost to stand‐replacing fire since 2001, with annual losses increasing through time in both the Americas and Africa. Protection most commonly reduced the proportion of stand‐replacing fire, but could not decouple temporal fire trends inside PAs from those outside.

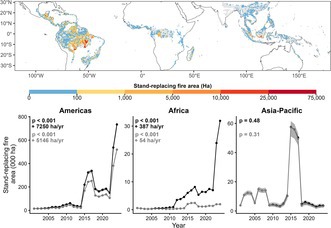

## 1 Introduction

Climate change is driving global shifts in temperature and rainfall regimes (Jolly et al. 2015), leading to increasingly frequent extreme events such as drought, heatwaves and wildfires (Seidl et al. 2017). Since 2001, fires alone have caused > 150 million hectares (Mha) of forest loss globally (Tyukavina et al. 2022). Annual fire‐driven forest loss has been increasing since the turn of the century, as has the frequency and intensity of extreme wildfire events, and weather conditions leading to elevated fire risk (Cunningham et al. 2024; Ellis et al. 2022; Tyukavina et al. 2022; Van Wees et al. 2021). Wildfires now account for ~30% of all forest loss at the global scale (Sims et al. 2025).

Tropical forests are global hotspots for biodiversity (Gibson et al. 2011; Pillay et al. 2022), vital stores of carbon (Pan et al. 2024), and provide food, timber and a wide range of ecosystem services that support the livelihoods of up to 1.5 billion people (Lewis et al. 2015). However, tropical moist forests are particularly vulnerable to fire damage due to low historical fire occurrence rendering tree species poorly adapted to fire, often resulting in high tree mortality rates post‐fire (Cochrane 2003). Concurrently, increasing forest fragmentation (Hansen et al. 2020; Taubert et al. 2018; Zou et al. 2025) and degradation (Engert et al. 2024; Lapola et al. 2023) are creating forest landscapes that are less resilient and more susceptible to fire (Cochrane 2003). Ensuring that tropical forests are effectively protected from adverse novel fire regimes now and into the future has thus never been more critical (Kelly et al. 2020).

A key element of historical and ongoing efforts to protect and maintain tropical forests and other natural habitats, that is enshrined in global agreements (e.g., Kunming‐Montreal Global Biodiversity Framework) (Bhola et al. 2021), is the provision and expansion of effective protected areas (PAs) (Butchart et al. 2010). There are currently > 300,000 protected areas covering > 16% of the global terrestrial land surface (UNEP‐WCMC and International Union for Conservation of Nature 2021), including > 2 million km^2^ of tropical forest (Sze et al. 2021). At the global scale, PAs reduce deforestation by ~41% (Wolf et al. 2021), with PA effectiveness varying by size, age, accessibility, population density and indigenous stewardship (Bowker et al. 2017; Leberger et al. 2020; Liu et al. 2022; Sze et al. 2021). PAs have suffered substantial levels of burning globally (Resco De Dios et al. 2025), but have been shown to reduce fire incidence compared to unprotected areas in certain regions (Biswas et al. 2015; Nelson and Chomitz 2011; Pessôa et al. 2023). The majority of fires in the tropics are caused by human activity either for intentional land clearance or the unintentional spread or escape of land management fires (Bowman et al. 2011; Cochrane 2003), and weather conditions conducive to wildfires are becoming more common (Jain et al. 2022; Jolly et al. 2015; Richardson et al. 2022). Whether tropical forest PAs are protected from the increasing risk of stand‐replacing fires across the tropical forest biome under increasingly favourable climate conditions, remains unknown.

Given their importance to tropical forest conservation, quantifying recent fire‐induced global losses of tropical forest inside PAs, and the relative susceptibility of PAs to the increasing fire frequency seen across the tropics, is critical for understanding their future effectiveness under global change. Here we combine high resolution global data of stand‐replacing fires (Tyukavina et al. 2022) with pan‐tropical PA extents and counterfactual modelling approaches to assess the past and ongoing efficacy of PAs in preventing fire‐induced tropical forest loss. We do so to answer four key questions: (1) How much protected tropical forest has been lost to stand‐replacing fires between 2001 and 2024 and where are these losses greatest? (2) What are the trends in annual fire‐driven losses of protected tropical forest? (3) Do PAs reduce fire‐driven forest loss compared to non‐PAs and (4) are PAs buffered from wider trends in the annual area of stand‐replacing fires compared to unprotected areas of tropical forest?

## 2 Materials and Methods

### 2.1 Data Sources

#### 2.1.1 Protected Areas

**FIGURE 1:**
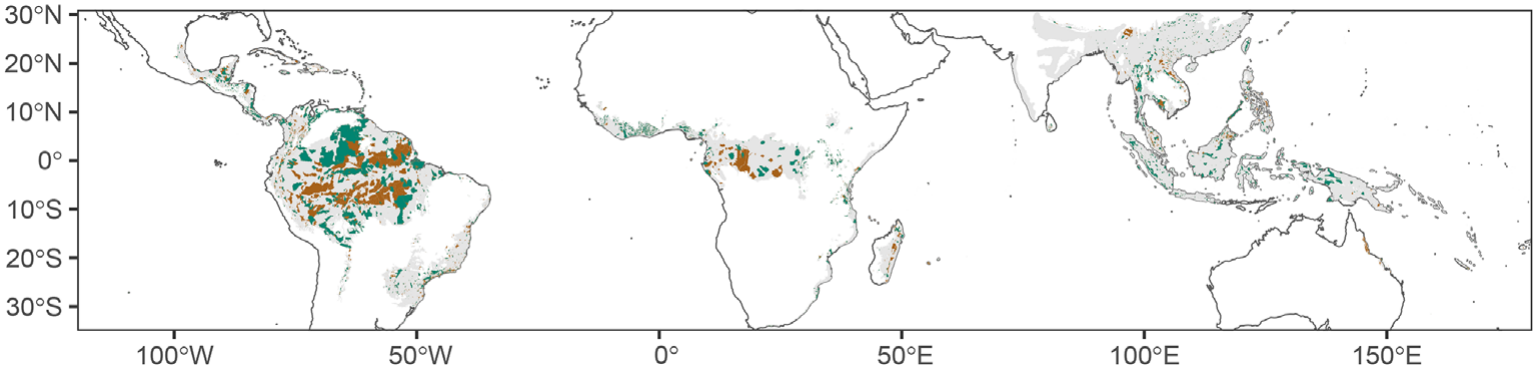
Map of the Protected Areas (PAs) used in this study. PAs were sourced from the World Database on Protected Areas (WDPA) and filtered for terrestrial PAs within the Tropical and Sub‐Tropical Moist Forest Biome. PAs coloured in green were designated prior to 2001, whereas those in brown were designated post 2001. Grey area represents the extent of the tropical and sub‐tropical moist forest biome (Dinerstein et al. 2017).

To estimate fire‐driven losses of tropical forest inside protected areas, we used the World Database on Protected Areas (WDPA) (UNEP‐WCMC and International Union for Conservation of Nature 2021). The WDPA is a joint project by the International Union for the Conservation of Nature (IUCN) and the United Nations Environment Programme (UNEP) and is the most comprehensive available dataset on the global distribution of protected areas. In this study, we used the July 2024 version of the WDPA which contained data on 283,894 protected areas globally. We considered all terrestrial PAs that were designated prior to 2024 and located within the tropical and sub‐tropical moist forest biome, excluding PAs where only point data were available (Dinerstein et al. 2017). Despite being the most comprehensive dataset available for the global distribution of PAs, data for some countries (e.g., China, India) are lacking due to restrictions on publicly available PA data. We included national‐level data from China by downloading PA data from the China Nature Reserve Specimen Resource Sharing Platform (http://www.papc.cn/), which included 313 PAs within the tropical and sub‐tropical moist forest biome. The final dataset used in the analysis therefore included 9791 protected areas (Figure 1). Publicly available and reliable data for India could not be sourced.

#### 2.1.2 Fire

For our measure of fire activity we used the dataset on forest loss caused by fire from Tyukavina et al. (2022). This dataset uses MODIS and Landsat imagery to map forest loss events (defined as the loss of woody vegetation > 5 m high) that were directly caused by stand‐replacing fires between 2001 and 2024, at a resolution of 30 m. We chose to use this dataset and not the MODIS Burned Area (Giglio et al. 2018) dataset employed in previous studies (Cardil et al. 2020; Resco De Dios et al. 2025) for two key reasons. Firstly, it is limited to stand‐replacing fires that cause a detectable loss of tree cover (and therefore forest habitat) and does not include small understory fires that degrade forests but do not necessarily lead to a complete loss of tree cover and forest habitat, and are more difficult to detect (Morton et al. 2013). Secondly, it provides significantly improved resolution (30 vs. 500 m) allowing for better distinguishing between forest fires and agricultural fires on forest borders. Overall, this choice of data will limit the extent of detected fire impacts compared to previous studies that use MODIS data, but ensures that reported areas of burn represent stand‐replacing wildfires where tropical moist forest cover has been lost. It therefore represents a reliable estimate of fire‐induced tree cover loss of tropical moist forest but will underestimate total fire‐induced impacts as it does not account for degradation associated with sub‐canopy fires. We compared the data with another 30 m resolution fire mapping data product (MapBiomas Fire Collection 5) to assess the accuracy of Tyukavina et al., finding good spatial agreement between the two products. When aggregating burned area within PAs to identical 5 km grid cells, the two datasets demonstrated a high level of spatial correlation (Pearson correlation coefficient of 0.74) across the Brazilian Amazon (Figure S1). The remaining discrepancy is likely due to MapBiomas also including understory fires alongside stand‐replacing events.

### 2.2 Fire‐Induced Loss of Tropical Forest Inside PAs

We crossed the protected area data with the fire data to estimate the total annual fire‐induced losses of tropical forest inside PAs. We used the 2000 tree cover data (Hansen et al. 2013) to consider only fire‐driven losses of forest where the tree cover in 2000 was ≥ 25% (the recommended threshold for identifying tall wooded vegetation unambiguously) (Hansen et al. 2013) and the forest was located within a protected area in the tropical and sub‐tropical moist forest biome (Dinerstein et al. 2017). We included a plantation mask using v2 of the Spatial Database on Planted Trees (Richter et al. 2024) to mask out any areas of plantation from our analysis. Where protected areas were designated after 2001 (i.e., the year our fire data is first available), we included in our analysis only fire events that occurred in the years following PA designation. We estimated total and annual fire‐induced losses of protected tropical forest in three tropical regions, Latin America, Africa and Asia‐Pacific, as well as aggregating losses by country according to the GADM country boundaries dataset (GADM 2022). Spatial overlays and area estimates were conducted using the sf (Pebesma 2016) and terra (Hijmans 2020) packages in R. When producing the map of forest loss due to fire, Tyukavina et al. (2022) undertook a sample‐based area estimate and ensured the final map of fire forest loss matched these area estimates for all regions, except for Africa. Additional versions of the map were produced for all regions except Africa that match the area estimate ±s.e.m. These maps were used to incorporate uncertainty in the form of s.e.m. to our estimates of tropical forest inside PAs lost to fire.

To assess the significance and size of temporal trends in annual forest loss due to fire inside PAs at the regional and national scale, we used Mann‐Kendall and Sen's slope tests from the trend package (Pohlert 2015). To account for the influence of new PAs being established post 2000, for trend analyses we only considered annual fire trends in a subset of PAs that were all established prior to 2001 (i.e., the year our fire data starts). We also tested trends across the entire PA estate inclusive of those established post‐2001.

### 2.3 Matching

We used statistical matching to assess whether tropical forests inside of protected areas suffered differing fire rates and temporal trends compared to forests outside of protected areas, whilst controlling for factors that likely influence fire activity and PA designation in tropical forests. To balance computational tractability with the need to capture small‐scale spatial heterogeneity (Duncanson et al. 2023; Sze et al. 2021), we aggregated the original fire and forest cover data (30 m resolution) up to 1 km plots, calculating for each 1 km plot the total forest (> 25% tree cover) area in 2000, and the total area of stand‐replacing forest fires in the period 2001–2024 (again masking out areas of plantation). For cells within PAs, we retained only those that were entirely bounded within the PA and had a tree cover > 25%. For cells outside PAs, we removed those that had a tree cover < 25%, or that were within a 10 km buffer of any PA to reduce spatial autocorrelation and limit edge or spillover effects from PAs. For this part of the analysis, we included only PAs that had been designated prior to 2001 (i.e., the year our fire data starts) to avoid introducing bias through the inclusion of areas that become protected in a later year.

Given the size of the dataset, we took a sample of observations from within and outside PAs for the matching analysis. For each region (Americas, Africa, Asia‐Pacific), we took a random sample of 250,000 forest cells within PAs, and 1.5 million forest cells outside of PAs to act as potential matches. We removed observations from countries where the total area of stand‐replacing forest fires across the sampled pixels for that country was < 0.1%. We used the MatchIt package (Ho et al. 2005) to undertake nearest‐neighbour matching for the following covariates: elevation (Amatulli et al. 2018), slope (Amatulli et al. 2018), mean annual temperature, mean temperature of the hottest quarter, mean annual precipitation, mean precipitation of the driest quarter and mean precipitation of the wettest quarter from the WorldClim dataset (Fick and Hijmans 2017), the 95th percentile of the Fire Weather Index based on ERA‐5 (Vitolo et al. 2020), population density (Center For International Earth Science Information Network‐CIESIN‐Columbia University 2017), travel time to nearest urban area of > 5000 people (Nelson et al. 2019), forest area inside the 1 km plot and forest area within a 5 km buffer of the forest plot (Hansen et al. 2013). We used exact matching for country. We conducted matching separately for each region and deemed a mean covariate balance of < 0.25 across all matched variables as acceptable. Following the advice of Schleicher et al. (2020), we tested matching with two different matching algorithms (Propensity Score Matching and Mahalanobis distance matching) with two different calipers (0.2, 0.5), and selected the best method based on the basis of mean covariate balance and retention of PA cells (Figure S2). Utilising this approach the most successful method was Mahalanobis distance matching with a calliper of 0.2 (which achieved mean SMDs of 0.01, 0.04 and 0.02 and variance ratios of 1.003, 0.957 and 0.967 in the Americas, Africa and Asia‐Pacific, respectively—see Figures S3–S5 for covariate distributions pre‐ and post‐matching). After matching, the dataset contained 49 countries (Americas = 23, Africa = 12, Asia‐Pacific = 14).

Though matching was conducted carefully, and to an acceptable standard, there remain some inevitable confounders that may influence results. For example, whilst we controlled for long‐term climate conditions, we cannot account for interannual or daily variations which have an important influence on fire ignition and spread potential. Similarly, data encompassing socio‐economic factors that could influence fire occurrence (e.g., fire management practices, local PA fire budgets, local governance) are not available at the necessary scales.

### 2.4 Modelling

While matching aims to reduce imbalance across all relevant covariates it does not eliminate it entirely. Thus, we conducted further regression adjustment to estimate the specific effect of PAs on the total proportion of stand‐replacing fire relative to non‐protected pixels between 2001 and 2024 (Figures S6 and S7).

We separately fitted GAMMs to each region's matched dataset after removing countries that had less than 250 post‐matching observations (Latin America *n* = 409,576, across 17 countries; Africa *n* = 268,602, 8 countries; and Asia Pacific *n* = 344,538, 11 countries). Prior to fitting we removed highly correlated matching variables, severely skewed covariates were log transformed using base 10 and all numeric covariates were mean centred and standardised. All continuous covariates were fit as cubic regression splines and status (protected, non‐protected) as a parametric effect. To incorporate the spatial distribution of sampling points we also included the effect of treatment as a spatially varying coefficient allowing independent smooths of protected status to vary across geographic latitude and longitude. Treating status as a spatially varying coefficient is essential as while we match on and include in regression adjustment a suite of covariates, there will be subnational variation in the effect of Protected Area driven by unknown variables and processes that it is not feasible to collect data on, for example, department and local administrative unit budget for relevant activities and local political will and corruption. We further included a hierarchical intercept for country and allowed the effect of status to vary by country to reflect that across space within countries we would hypothesise the existence of a broad consensus effect. All models were fitted with quasibinomial error structure to reflect the increased dispersion not effectively modelled with a binomial structure. In all models the response was defined as the proportion of burned 30 m cells out of the total number of forested 30 m cells per 1 km grid.

We use the parametric g‐formula approach to estimate the counterfactual effect of protected‐area status, for those areas currently protected (the average treatment effect on the treated, ATT), on fire occurrence. This specifically asks if currently protected areas burn differently due to protection. In this approach the fitted model is used to estimate the predicted burn probabilities across the observed dataset of protected 1 km cells and all observed covariate values having fixed the status variable as “protected” this is then repeated but each pixel's status is switched to ‘non‐protected’. Thus, this approach directly asks what the effect of all protected matched pixels being protected has on estimated burn probabilities. We defined the counterfactual difference in two ways, firstly as the simple difference (the percentage point change from protected to non‐protected), and secondly as the percentage difference between protected and non‐protected (the ratio of burn probabilities in protected and non‐protected plots). The former is presented in the main text, and the latter in the Supporting Information.

We further fitted an additional temporal model per region to specifically ask if equivalent protected areas are decoupled from the wider pattern of increasing stand‐replacing forest fire area over the last 24 years. This used the same matched pixels as in the previous analysis but tracked their stand‐replacing forest fire area annually yielding the number of forest pixels in each plot lost to stand‐replacing fire each year. Likewise, we also calculated the remaining forest pixels annually ensuring the proportion of forest burnt each year reflected the loss of any forest to fire in previous years. This resulted in a disaggregated temporal dataset (Latin America *n* = 9,829,788, Africa *n* = 6,446,448, Asia Pacific *n* = 8,268,211). The covariate structure detailed for the previous model was retained, including the spatially varying coefficient on protected status. We additionally included separate smooths for protected status through time, not‐constraining protected and non‐protected areas to the same temporal function. We also further included the hierarchical effect of protected status through time, per country. All models were checked for convergence and fit, examination of model residuals revealed good fit, although a slight underestimation of extremely severe fire events (e.g., where more than 50% of a cell burnt), however such events make up ~1%, < 0.01%, and ~0.9% of observations for Latin America, Africa and Asia Pacific, respectively.

To isolate the temporal trends between protected and not protected areas we set all covariates to their means and excluded the smooth on spatial coordinates and country, thus focusing on the temporal trend for the average per region. From this trend we extracted the annual marginal effect (slope) for protected and unprotected areas.

All models were fit and checked with the ‘mgcv’ package (Wood 2000) and all post‐fitting handling, prediction and comparison conducted with the ‘marginaleffects’ (Arel‐Bundock 2021) package.

## 3 Results

### 3.1 Extensive Pan‐Tropical Losses of Protected Forest due to Fire

**FIGURE 2:**
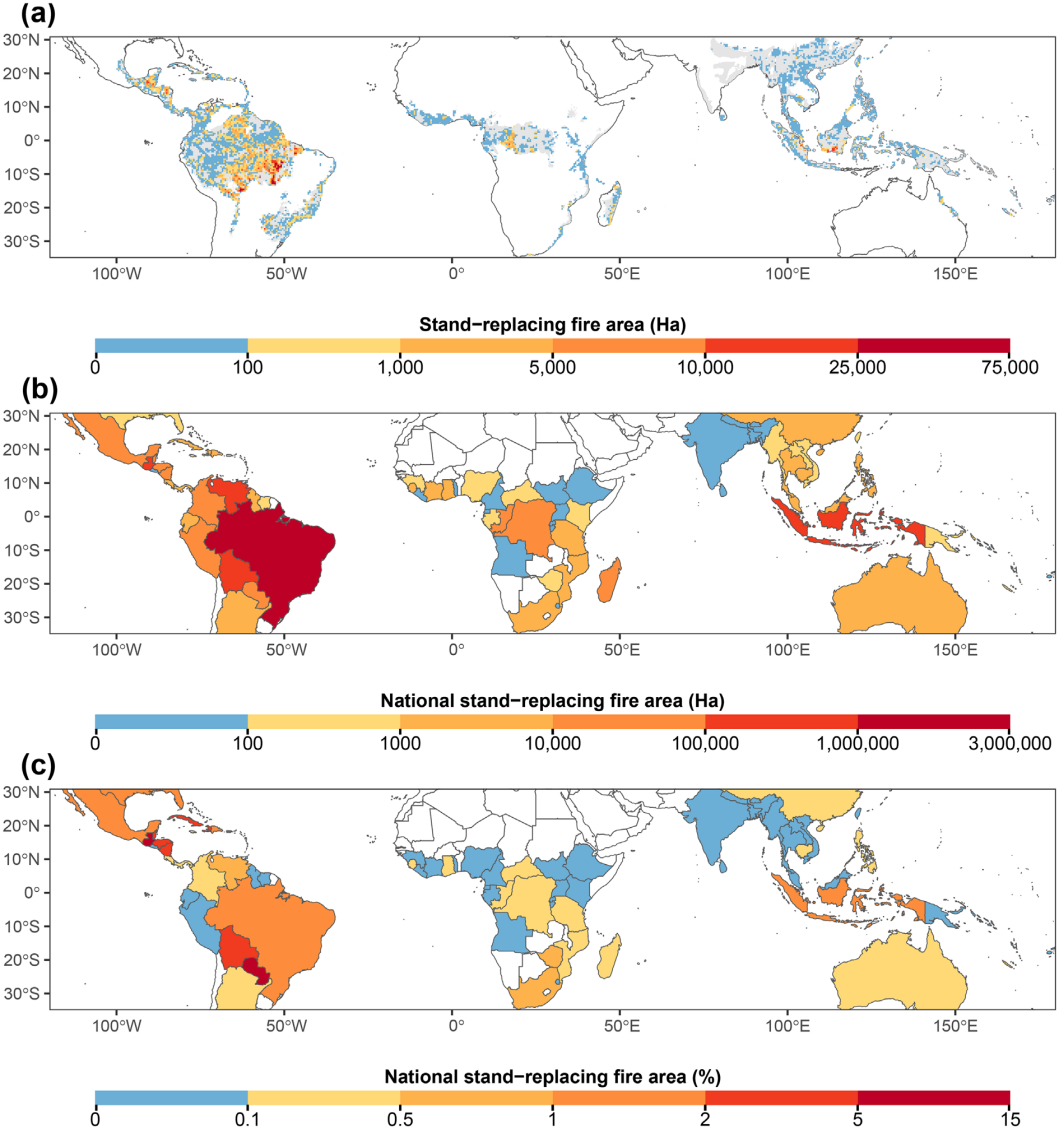
Pan‐tropical burning of protected tropical forest between 2001 and 2024. Shown is the total area (Ha) of all protected tropical forest lost to stand‐replacing wildfire in each 50 km^2^ cell (a), the total area of protected tropical forest lost (Ha) at the national scale (b), and the percentage of all protected tropical forest lost at the national scale (c). Map lines delineate study areas and do not necessarily depict accepted national boundaries.

Between 2001 and 2024, 4.07 (s.e.m. 3.77–4.31) Mha of tropical forest inside PAs suffered stand‐replacing fires (Figure 2a), representing a global loss of 1% of all tropical forest inside PAs in just over 20 years. The vast majority (89%) of fire‐induced losses were observed in the tropical Americas (3.62 Mha, 3.37–3.83 Mha), followed by Asia Pacific (0.31 Mha, 0.26–0.35 Mha) and Africa (0.14 Mha). Hotspots of fires inside tropical forest PAs were concentrated in Central America, the Southern Amazon, the Brazilian Atlantic Forest and Kalimantan and Sumatra (Figure 2a, see Figure S8 for more detailed maps).

At the national scale, Brazil experienced the largest area of fire‐induced tropical forest loss inside PAs (Figure 2b) at 2.66 (2.51–2.78) Mha, accounting for 65% of global losses, followed by Bolivia (0.39 Mha, 0.36–0.41 Mha) and Indonesia (0.28 Mha, 0.24–0.31 Mha). This represents a fire‐induced loss of 1.3%, 4.3%, and 1.6% of national protected tropical forest in each of these countries, respectively (Figure 2c, see Table S1 for national estimates). Proportionally the greatest fire‐driven losses of protected tropical forest were in Paraguay (12.6%, 0.04 Mha), Guatemala (6.7%, 0.1 Mha) and Haiti (4.7%, 0.001 Mha).

At the PA scale, 4 PAs lost > 50% of their forest to stand‐replacing fires between 2001 and 2024, 18 PAs lost > 25% and 95 PAs lost > 10%. The majority of PAs that lost > 10% of their forest were located in the Americas (69), with 23 in Asia‐Pacific and 3 in Africa. The management category of the PAs (strict, multi‐use or unknown) had little impact on the proportion of forest lost to wildfire. Similarly, under the average regional conditions, increasing PA size and greater surrounding forest cover had no clear association with changes in stand‐replacing wildfires (Methods S1 and Figure S9).

### 3.2 Increasing Annual Losses of Protected Tropical Forest

**FIGURE 3:**
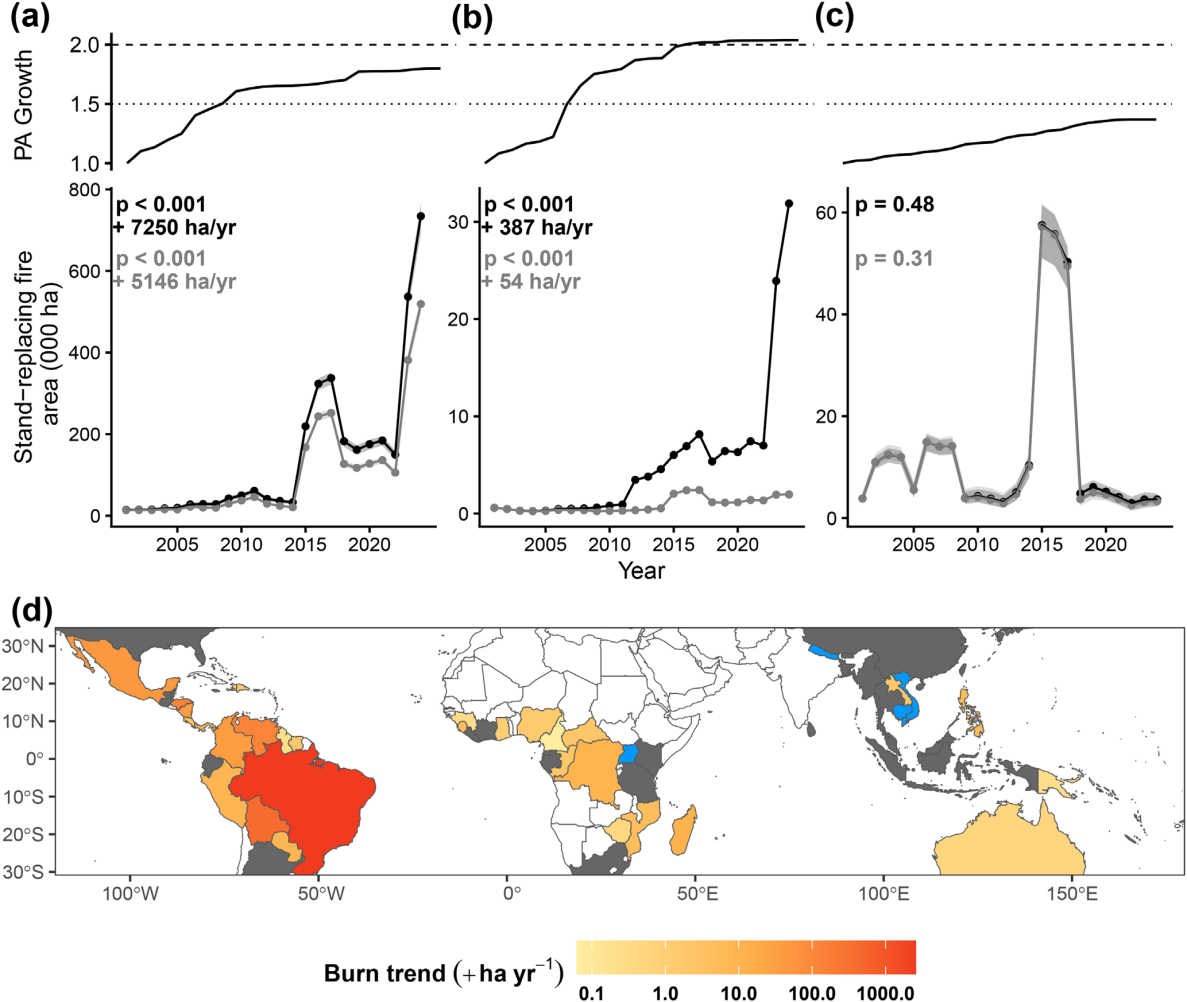
Temporal trends of stand‐replacing fire in protected tropical forest between 2001 and 2024. Shown from left to right for Latin America (a), Africa (b) and Asia‐Pacific (c) is the total ‘PA Growth’ for each region (i.e., relative change in PA area compared to 2000), and the 3‐year average annual area of protected tropical forest lost to wildfires across all PAs, including those established post 2001 (black), and only PAs designated prior to 2001 (grey). See Figure S10 for an annual version, and Figure S11 for proportion lost per year. Shown in (d) is the size of the national‐level trend in stand‐replacing fire area between 2001 and 2024 for PAs designated prior to 2001. Inset text in (a–c) denotes the average annual burn trend and significance derived from Mann‐Kendall tests, dotted line represents 50% increase, dashed line represents 100% increase in PA estate. In (d), dark grey denotes no significant trend, blue denotes decreasing trends, and shades of yellow to red represent progressively large increasing trends. Map lines delineate study areas and do not necessarily depict accepted national boundaries.

Across the tropics, fire‐induced losses of tropical forest inside protected areas demonstrated significant increasing trends between 2001 and 2024 (Mann‐Kendall test, *p* < 0.001). This remains the case even when accounting for expansion in the tropical forest PA network throughout this century, which has grown by 80%, 104% and 37% since 2000 across the Americas, Africa and Asia‐Pacific, respectively (Figure 3). At the regional scale, there were strong increasing trends across the Americas (Mann‐Kendall test, *p* < 0.001) and Africa (*p* < 0.001), but no trend in Asia‐Pacific (*p* = 0.31). This remains true when only considering PAs inscribed prior to 2001, although increasing trends are reduced slightly, particularly in Africa. Nevertheless, the mean annual area of stand‐replacing forest fires inside PAs has increased more than ninefold since 2016 compared to the annual average between 2001 and 2015 (when considering only PAs designated prior to 2001, the increase is fivefold). In 2024 alone, 1.35 Mha of tropical protected forest was lost to fire (one third of all losses in the period), with 95% of this loss occurring in the tropical Americas (Figure S10). Despite the massive extent of fire‐induced forest loss in 2024, it did not influence the temporal trends, which demonstrated a significant increase in the Americas and Africa when considering only 2001–2023 (Table S2).

When controlling for PA growth between 2001 and 2024 by considering only PAs designated prior to 2001, significant increasing trends in annual fire‐driven losses of tropical forest inside PAs were detected in 30 countries across the tropics, 15 of which were in the Americas, 11 in Africa and 4 in Asia‐Pacific (Figure 3d). Countries exhibiting increasing trends in stand‐replacing forest fires included four of the five countries with the largest extent of tropical forest inside PAs (Brazil, Venezuela, Peru, DRC), whilst only five countries (Uganda, Nepal, Vietnam, Cambodia, and Trinidad & Tobago) exhibited decreasing trends and 27 countries showed no trend. In combination, countries exhibiting increasing trends in annual stand‐replacing forest fires host 80% of all protected tropical forest globally. Additionally, incorporating burning in PAs designated after 2001 shows highly comparable national trends (Figure S12).

### 3.3 Contrasting Global Impacts of Protection on Fire Probability

We compared the proportion of stand‐replacing fire in matched 1 km forest plots inside and outside of PAs using statistical matching and hierarchical spatially‐varying‐coefficient models to control for confounding variables and sub‐national variation over the last 23‐years (e.g., proximity to anthropogenic activities, weather patterns and topography).

**FIGURE 4:**
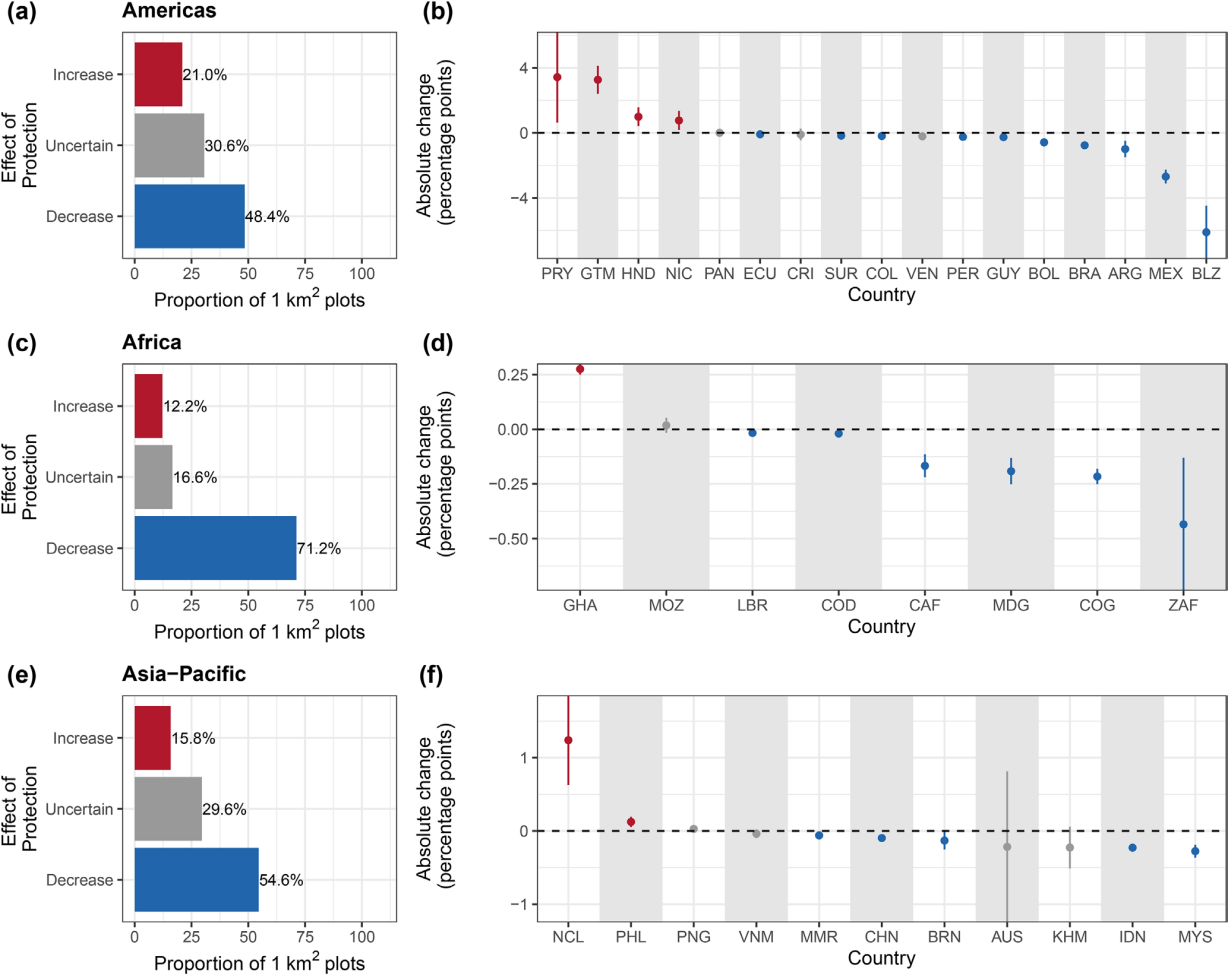
Counterfactual contrasts in the proportion of stand‐replacing fire in protected and non‐protected areas. Shown is the counterfactual summary for the average treatment effect for the treated (ATT) direction for each plot in the Americas (a), the country level counterfactual ATT estimates for the effect of protection summarised to the absolute change (percentage points) for the Americas (b), the same is shown for Africa in (c) and (d), and Asia‐Pacific in (e) and (f). In all panels colour denotes a decreased proportion of stand‐replacing fire (blue), uncertain effect (grey) or increased due to protection (red). Points are means and error bars 95% confidence intervals. See Figure S13 for percentage differences as opposed to percentage point change and Figure S14 for individual plot estimates. See Table S3 for a full list of three letter ISO codes and their corresponding country name in full.

In the Americas, we estimate that protection reduced the proportion of stand‐replacing fire in 48.4% of protected plots (Figure 4a) and increased the proportion for 21.0%, with the remaining plots showing no clear difference (Figure 4a). In more than half of the countries in the Americas (10/17), protected areas had a reduced proportion of stand‐replacing fire. However, despite their relatively small protected area estate, protected forest in Paraguay and Guatemala respectively had an 3.42 percentage point (95% CI: 0.63–6.22 pp, Figure 4b) and 3.27 pp (2.41–4.13 pp) increase in the absolute proportion of stand‐replacing fire compared to unprotected forest (a relative increase of 29% and 62%, Figure S13).

Across Africa protected plots were predominantly less likely to burn (71.2%) and saw an increased proportion of stand‐replacing fire in only 12.2% of plots (Figure 4c). Six out of the eight African nations assessed (South Africa, the Republic of the Congo, Madagascar, the Central African Republic, the Democratic Republic of the Congo and Liberia) showed that protection decreased the proportion of stand‐replacing fire, with increases only likely in Ghana (Figure 4d). Across Asia‐Pacific, protected plots had similarly reduced proportions of stand‐replacing fire (54.6%, Figure 4e), with increased burning only estimated in 15.8% of plots. At the national level, protection had clear average reductions in the proportion of stand‐replacing fire in 5 out of 11 countries (Figure 4f). In biodiversity hotspots such as Indonesia and Malaysia, protection is associated with a small 0.23 pp (−0.27 to −0.19 pp) and 0.28 pp (−0.37 to −0.19 pp) decrease in the absolute proportion of stand‐replacing fire, respectively (a 12% and 83% relative decrease, Figure S13).

### 3.4 Limited Decoupling From Changing Tropical Fire Regimes in PAs

**FIGURE 5:**
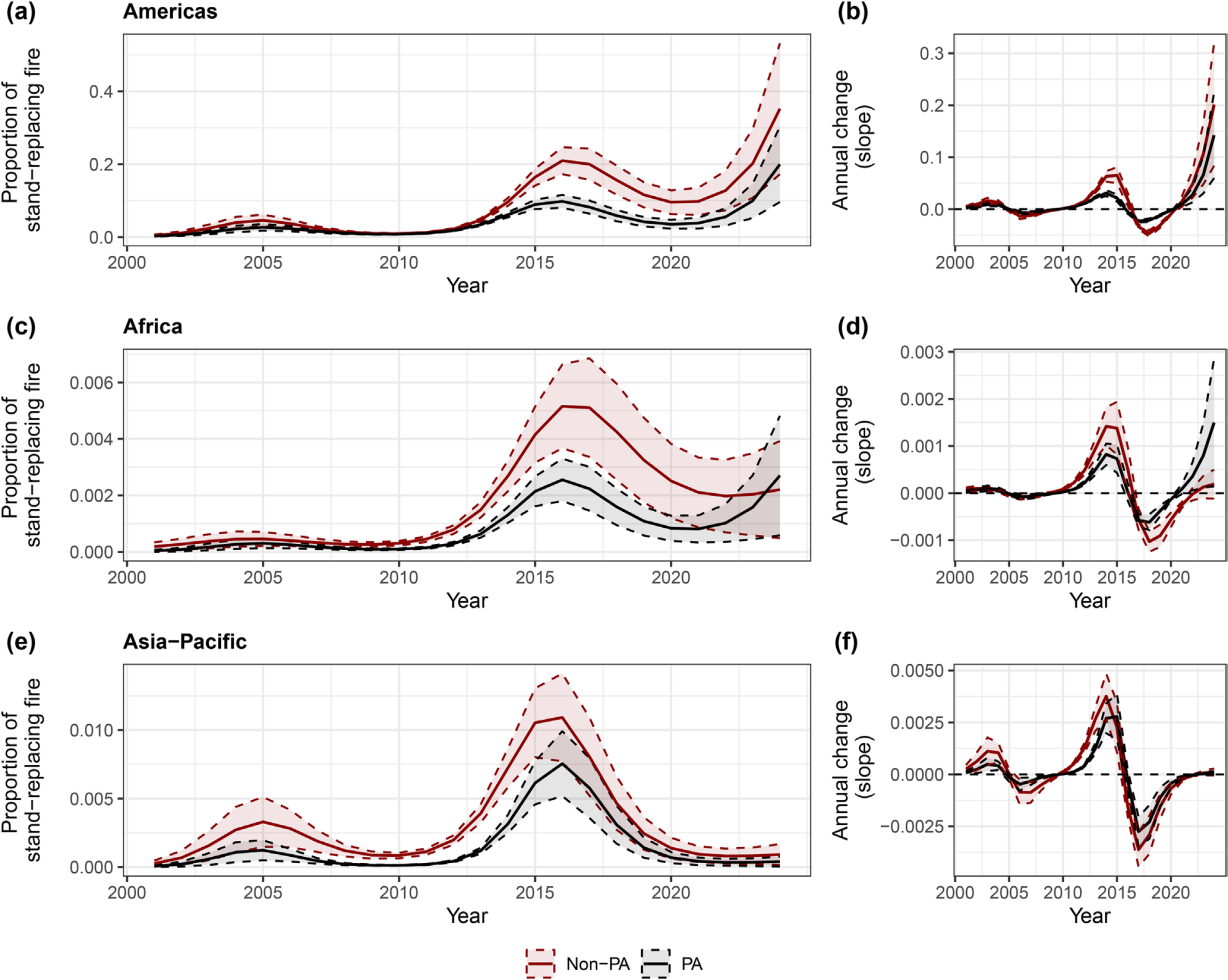
Temporal trends in the proportion of stand‐replacing fire for the average plot in protected and non‐protected areas. Shown are the temporal trends in burnt percentage for the average protected and non‐protected plot in Latin America (a), the marginal effect (slope) each year in the average protected and non‐protected plot in Latin America (b); this is repeated for Africa in (c) and (d), and Asia‐Pacific in (e) and (f). Solid lines represent the predicted means and the shaded ribbons the 95% confidence interval.

To assess whether PAs were protected from general increases in fire activity compared to non‐PAs, we compared annual trends in the proportion of stand‐replacing fire in protected tropical forests with trends in matched unprotected forests in the same counterfactual modelling framework. Across all regions, forests inside PAs demonstrate highly similar trends in annual stand‐replacing forest fire compared to unprotected forest, suggesting that PAs are not protected or decoupled from temporal changes in fire regimes (Figure 5). In the Americas, protected and unprotected areas average trends only differed in direction once over 24 years (Figure 5b), in Africa they differed in 5 years (Figure 5d) and in Asia‐Pacific they only did so once (Figure 5f). Strong inter‐annual variability in the proportion of stand replacing forest fires is mostly driven by large‐scale climate patterns (e.g., El Nino droughts in 2015–2016 and 2023–2024) (Burton et al. 2020; Signori et al. 2026), with forests both inside and outside of PAs showing similar susceptibility to these processes.

This lack of decoupling from increasing stand‐replacing fire trends is particularly concerning in the Americas, which has the majority of tropical forest lost to fire, the clearest increasing trend in stand‐replacing fire through time, and relatively small differences in the temporal proportion of stand‐replacing fire inside and outside of the average PA (Figure 5a,b, Figure S15). Likewise, whilst the proportion of stand‐replacing fire in Africa is comparatively low, and predominantly lower inside PAs than outside until 2022 (Figure 5c, Figure S15b), trends in the proportion of annual stand‐replacing forest fire remain highly similar and both demonstrate significant increases (Figure 5d). Whilst not demonstrating increasing trends, forests inside and outside of PAs in Asia‐Pacific also exhibit the same shifts in stand‐replacing fire between years. This suggests that while PA status may reduce the proportion of stand‐replacing fire in some regions, PAs remain tightly coupled to wider shifts in fire regimes.

Our findings come with four key caveats. First, the fire data used accounts only for stand‐replacing fire events and does not include sub‐canopy, lower intensity fires that do not cause substantial tree cover loss at the pixel scale. Such fires are widespread in the tropics (Morton et al. 2013), accounting for a similar number of fire events as stand‐replacing fires in the Amazon in 2020 (East et al. 2023), and can have severe ecological consequences (Barlow et al. 2003; Barlow and Peres 2008). These fires are not represented in this study, meaning the fire impacts presented here will be an underestimate. Second, advancements in forest loss detection capabilities (Hansen et al. 2013) over the period covered by our study necessitate caution when comparing trends between the 2000–2010 and 2011–2024 period. However, whilst we detect increasing trends in the area of stand‐replacing forest fires in tropical PAs across the entire period (2001–2024), we also detect the same increasing trends when limiting our analysis to 2011–2024, during which forest loss detection capabilities were consistent. The fire data used (Tyukavina et al. 2022) were also tested against fire forest loss detection algorithms that were consistent across 2001–2024 and demonstrated the same trends (Tyukavina et al. 2022). Third, by restricting our analysis to the tropical and sub‐tropical moist forest biome we limit the inclusion of tropical dry forests which possess their own, non‐pyrophobic fire ecology. However, in areas where these dry and moist forests co‐occur detrimental losses may be overestimated (Williamson et al. 2024). Finally, we estimate fire‐driven forest loss but cannot explicitly distinguish between different causes of fire (e.g., deliberate deforestation fires and uncontrolled wildfires), which will be differentially important between regions and require contrasting management strategies. Likewise, we do not explicitly separate edge effect fires from other fire types within PAs and thus may underestimate their impacts (Armenteras et al. 2013).

## 4 Discussion

Between 2001 and 2024, more than 4 Mha of protected tropical forest were lost due to fires, with increasing trends in annual stand‐replacing fires in the tropical Americas and Africa. Forests inside PAs most often had a lower proportion of stand‐replacing fire than forest outside of PAs (although we observed substantial national heterogeneity), emphasizing the role PAs can have in global strategies to conserve biodiversity. However, trends in annual stand‐replacing fires over the preceding 24 years have been closely coupled, demonstrating that PA status currently does not clearly decouple tropical forests from changing fire regimes. Given the expected increase in fire frequency and intensity in the future (Bowman et al. 2020) alongside global commitments to expand the protected area estate (Dinerstein et al. 2019), vulnerability to future wildfires must be a critical consideration in both ongoing management and future expansion of the tropical PA network.

Our findings of 4.07 Mha of protected tropical forest lost to fire is substantial, but significantly lower than other values reported in the literature. A recent global analysis suggests that burning occurred across 88 Mha of tropical moist forest PAs (Resco De Dios et al. 2025) between 2001 and 2024, whilst others found that 6.9 Mha of the Amazon Basin (including non‐PA area) burned in 2019 alone (Cardil et al. 2020). These discrepancies are primarily due to the choice of datasets used. Here we use the fire‐induced forest loss data from Tyukavina et al. (2022). Most other studies use MODIS or VIIRS burned area products which lead to far higher estimates due to three key reasons. Firstly, they include understory fires that do not result in a loss of tree cover. Such fires are extensive across the tropics (Morton et al. 2013), and can cause significant degradation (Barlow et al. 2003; Barlow and Peres 2008) but often not a loss of forest cover. Secondly, the fire data used here is limited to areas where fire directly caused the loss of tree cover and therefore does not include areas that were deforested and subsequently burned to prepare the land for alternative use. Such fires accounted for > 85% of burned area in the Amazon in 2019 (Cardil et al. 2020) but are not included in this analysis as fire was not the primary driver of forest loss. Finally, the high‐resolution (30 m) of the data used here (Tyukavina et al. 2022) allows for more effective distinction between fires occurring on forested land and agricultural fires occurring in land adjacent to forest, which is not effectively determined by lower‐resolution products such as MODIS (500 m) and VIIRS (375 m). In sum, our estimates represent a lower bound of fire damage, focusing on areas that suffered the most extreme fires and a total loss of tree cover, which could take decades to re‐establish, if at all (Barlow et al. 2018; Barlow and Peres 2008; Veldman and Putz 2011). The total impact of fire in tropical moist forest PAs is likely far larger once accounting for the degradation associated with understory fires.

Widespread and increasing stand‐replacing fire across protected tropical forests is a substantial threat to the ecosystem services they provide. Many regions of tropical forest lack historical exposure to large fires (Cochrane 2003) and are susceptible to cascading compositional turnover (Barlow and Peres 2008). This can trigger state shifts towards fire‐prone grass‐dominated savannah systems (Barlow et al. 2018; Veldman and Putz 2011) and the loss of the globally vital biodiversity and carbon supported by tropical forests (Mitchard 2018; Pillay et al. 2022). The strong increases in fire activity observed here are likely a combination of growing anthropogenic pressure alongside climate‐induced increases in conditions conducive to wildfire activity (Jain et al. 2022). Shifting climate conditions are increasing the fuel load and dryness in tropical moist forests which predisposes them to historically rare fire events (Cochrane 2003), particularly in the Amazon, which has seen some of the strongest recent increases in extreme weather conditions conducive to fire (Jain et al. 2022). Concurrently, high deforestation rates are causing region‐wide drying (Barkhordarian et al. 2019), while forest degradation (e.g., logging, road construction, fragmentation) exacerbates these changes, creating hotter, drier canopy gaps and increased fuel loads (Lindenmayer et al. 2020; Machado et al. 2025).

Given that a substantial portion of forests inside PAs exhibited lower burn rates than forests outside of PAs across the tropics, it is likely effective management of burn risk inside tropical PAs can reduce fire occurrence, further underscoring the need for nuanced, context specific fire management. As the majority of fires in the tropics are started by humans (Bowman et al. 2011) either deliberately to clear land (e.g., for pasture or agriculture) or accidentally through the spread or escape of land management fires (Barlow et al. 2020), managing anthropogenic pressures is vital. Where fires are used for deliberate land clearance inside PAs, stronger local enforcement and monitoring are needed to prevent encroachment (Wolf et al. 2021). Conversely, working with local communities to reduce the unintentional spread of agricultural fires must be incorporated into management plans (Barlow et al. 2020). Such fires are essential to rural food security (Carmenta et al. 2013), but can account for > 50% of burning inside PAs (Román‐Cuesta and Martínez‐Vilalta 2006). Public fire mitigation programmes that provide technical and fire management assistance to landowners can reduce burned areas by up to 35% (Oliveira et al. 2021). Greater emphasis and investment in fire prevention are also vital. In the Brazilian Amazon, fire management budgets average US$0.51 ha^−1^ year^−1^ and only 6% of this is spent on prevention (Oliveira et al. 2021). Furthermore, improved fire modelling techniques could allow for better spatial prediction of fire ignition (Gora et al. 2020), whilst automated fire detection and suppression approaches such as drone‐based infrared sensors, ground‐based sensors or automated cameras on fire towers could allow for 24‐h fire detection and suppression (Lindenmayer et al. 2023). Increasing future fire risk in tropical forests will require significant increases in economic resources allocated to both fire prevention and suppression.

Tropical forests are already experiencing novel climate and fire regimes as a result of anthropogenic climate change, and will continue to do so in the future (Trew, Edwards, et al. 2024). Existing protected areas do not appear to be decoupled from these wider climatic shifts, and subsequent trends in forest fire occurrence. It is therefore critical to ensure that the future designation of protected areas considers changing fire risk and forest viability under climate change (Anderegg et al. 2020). Understanding which characteristics cause reduced fire activity inside individual PAs (e.g., size, connectivity, management) is a critical question for future PA design. Furthermore, embedding consideration of habitat robustness into conservation area planning has already been suggested for Key Biodiversity Areas and prioritising protection of those areas that are buffered from ongoing climatic novelty (Trew, Lees, et al. 2024), including fire risk. This is of critical importance across the Americas in particular, where future burned area is likely to be driven by increasing dryness and thus potentially less likely to be mitigated by altered PA management (Wu et al. 2021).

The growing loss of protected tropical forest to fire and the incomplete effectiveness of PAs—especially in the Americas—in reducing this loss underscores the critical need for the current and future PA estate to embed robust considerations and mitigation strategies for fire risk. In 2024 alone, a record breaking 1.35 Mha of protected tropical forest was lost to fires, with devastating consequences for biodiversity, forest ecosystem services and human health. A concerted international effort from designation to implementation and management is therefore needed to ensure that such losses are the exception and do not become the rule.

## Author Contributions

**Christopher G. Bousfield:** writing – original draft, methodology, conceptualization, investigation, writing – review and editing, formal analysis. **Oscar Morton:** formal analysis, writing – review and editing, methodology, conceptualization, investigation, writing – original draft.

## Funding

O.M. received financial support from the Leverhulme Trust (ECF‐2024‐005).

## Conflicts of Interest

The authors declare no conflicts of interest.

## Supporting information


**Figure S1:** Spatial comparison between stand‐replacing fires mapped by Tyukavina et al. and burn scars mapped by MapBiomas.
**Figure S2:** Standardised mean differences from matching results testing the impact of matching algorithm (PSM, Mahalanbodis) and caliper size (0.2, 0.5).
**Figure S3:** Covariate distribution for unmatched and matched datasets.
**Figure S4:** Covariate distribution for unmatched and matched datasets.
**Figure S5:** Covariate distribution for unmatched and matched datasets.
**Figure S6:** Summary of raw matched data.
**Figure S7:** Predicted counterfactual burnt percentage.
**Figure S8:** Pan‐tropical burning of protected tropical forest between 2001 and 2024.
**Figure S9:** Predicted probability of fire across PA characteristics.
**Figure S10:** Temporal trends of stand‐replacing fire in protected tropical forest between 2001 and 2024.
**Figure S11:** Temporal trends of stand‐replacing fire in protected tropical forest between 2001 and 2024.
**Figure S12:** National‐level temporal trends of stand‐replacing fire in protected tropical forest between 2001 and 2024.
**Figure S13:** Counterfactual contrasts in the probability of stand destroying fire in protected and non‐protected Areas (as in Figure 3 but showing % change rather than percentage point).
**Figure S14:** Counterfactual contrasts across the protected area network.
**Figure S15:** Contrasted burnt area through time for the average Protected and Non‐Protected plot.
**Table S1:** National level estimates of stand‐replacing wildfires in tropical forests in Protected Areas.
**Table S2:** Impact of removing 2024 from trend analysis.
**Table S3:** ISO‐codes and corresponding country names used in this study.

## Data Availability

All data used in this study is freely available for download online. The stand‐replacing fire data is available at: https://glad.umd.edu/dataset/Fire_GFL/, the protected area data is available at: https://www.protectedplanet.net/en/thematic‐areas/wdpa?tab=WDPA, the tree cover data is available at: https://storage.googleapis.com/earthenginepartners‐hansen/GFC‐2024‐v1.12/download.html. The code for the analysis is available at https://doi.org/10.5281/zenodo.21822098.
