# Supplementary material for: Escalating Fire‐Driven Forest Loss in Protected Tropical Forests
Source: Glob Chang Biol. 2026 Aug 11;32(8):e71051. doi: 10.1111/gcb.71051 (PMC13462366; doi:10.1111/gcb.71051)
Supplement: Supplementary file 1 [file GCB-32-e71051-s001.docx]

**Supplementary Materials - Escalating fire-driven forest loss in protected tropical forests**

Christopher G. Bousfield^1^*^†^ & Oscar Morton^1^*^†^

**Affiliations:**

^1^ Ecology and Evolutionary Biology, School of Biosciences, the University of Sheffield, S10 2TN, UK.

* Correspondence: c.bousfield@sheffield.ac.uk, [o.morton@sheffield.ac.uk](mailto:o.morton@sheffield.ac.uk)

^†^ These authors contributed equally to this work

**Supplementary Methods**

To explore how the proportion of stand destroying fire varied across PAs we focused on three key predictors, protection type, PA size, and the proportion of tropical forest within 50km. We simplified the 7-level protection classification to “Multi-use” (Categories V and VI) and “Strict” (Categories Ia, 1b, II, III, and IV) and “Unknown”. We modelled the proportion of stand destroying fire (2000-2024) per region for all PAs established before 2001 (the Americas n = 1720, Africa n = 1389, Asia-pacific n = 1132) using quasibinomial generalised additive mixed models (GAMMs). We included continuous covariates likely to affect fire heterogeneity (travel time, elevation, topographic slope, population density, precipitation, the fire weather index), which were fit as cubic regression splines. A smooth of geographic latitude and longitude, and hierarchical intercepts per country were also included. Our focal variables were included as parametric effects (protection type) and splines (extent of the protected area the plots fall within and the proportion of tropical forest within a 50km of that plot).

**Figure S1.** **Spatial comparison between stand-replacing fires mapped by Tyukavina *et al.* and burn scars mapped by MapBiomas.** Shown is the total area per 5km^2^ cell of mapped stand-replacing fires (Tyukavina *et al.* 2022) within PAs used in this study (a), and burn scars detected by MapBiomas Fire Collection 5 (b). The two products had a Pearson-correlation coefficient of 0.74, with MapBiomas mapping three times greater fire area due to its inclusion of non-stand-replacing understory fires.

**Figure S2. Standardised mean differences from matching results testing the impact of matching algorithm (PSM, Mahalanbodis) and caliper size (0.2, 0.5).** Red dots represent the standardised mean difference (SMD) between treatment (PA) and control (non-PA) 1km forest plots prior to matching, and blue dots post matching. Dashed line represents a SMD of 0.25

**Figure S3. Covariate distribution for unmatched and matched datasets.** Shown are the distribution density for each covariate before and after the matching process for the Americas.

**Figure S4. Covariate distribution for unmatched and matched datasets.** Shown are the distribution density for each covariate before and after the matching process for Africa.

**Figure S5. Covariate distribution for unmatched and matched datasets.** Shown are the distribution density for each covariate before and after the matching process for Asia-Pacific.

**Figure S6. Summary of raw matched data.** a, show Latin America, b, show Africa and c, Asia. Points are means and intervals are the 2.5 and 97.5% quantiles.

**Figure S7. Predicted counterfactual burnt percentage.**  a, show Latin America, b, show Africa and c, Asia. Points represent the predicted mean and the error bars the 95% confidence intervals.

**Figure S8. Pan-tropical burning of protected tropical forest between 2001 and 2024.** Shown is the total proportion of all protected tropical forest lost to wildfire in each 50km2 cell across the Americas (a), Africa (b), and Asia-Pacific (c).

**Figure S9. Predicted probability of fire across PA characteristics.** Proportion of stand replacing fire in the Americas across protection types (a), PA area (b) and the proportion of forest within 50km of the plot (c) for the average PA. The same is shown across Africa (d, e and f) and Asia-Pacific (g, h, and i). All solid lines and points are means, error bars and uncertainty ribbons are the 95% confidence intervals.

**Figure S10. Temporal trends of stand-replacing fire in protected tropical forest between 2001 and 2024.** Shown from left to right for Latin America (a), Africa (b) and Asia-Pacific (c) is the total ‘PA Growth’ for each region (i.e. relative change in PA area compared to 2000), and the annual area of protected tropical forest lost to wildfires across all PAs, including those established post 2001 (black), and only PAs designated prior to 2001 (grey). Shown in (d) is the size of the national-level trend in stand-replacing fire area between 2001 and 2024 for PAs designated prior to 2001. Inset text in (a, b, c) denotes the average annual burn trend and significance derived from Mann-Kendal tests, dotted line represents 50% increase, dashed line represents 100% increase in PA estate. In (b), dark grey denotes no significant trend, blue denotes decreasing trends, and shades of yellow to red represent progressively large increasing trends.

**Figure S11. Temporal trends of stand-replacing fire in protected tropical forest between 2001 and 2024.** Shown from left to right for Latin America (a), Africa (b) and Asia-Pacific (c) is the total ‘PA Growth’ for each region (i.e. relative change in PA area compared to 2000), and the annual proportion of protected tropical forest lost to wildfires across all PAs, including those established post 2001 (black), and only PAs designated prior to 2001 (grey). Shown in (d) is the size of the national-level trend in stand-replacing fire area between 2001 and 2024 for PAs designated prior to 2001. Inset text in (a, b, c) denotes the average annual burn trend and significance derived from Mann-Kendal tests, dotted line represents 50% increase, dashed line represents 100% increase in PA estate. In (b), dark grey denotes no significant trend, blue denotes decreasing trends, and shades of yellow to red represent progressively large increasing trends.

**Figure S12. National-level temporal trends of stand-replacing fire in protected tropical forest between 2001 and 2024.** Shown is the size of the national-level trend in stand-replacing fire area inside PAs between 2001 and 2024 across all PAs, including those designated post 2001. Dark grey denotes no significant trend (Mann-Kendall test), blue denotes decreasing trends, and shades of yellow to red represent progressively large increasing trends.

**Figure S13. Counterfactual contrasts in the probability of stand destroying fire in protected and non-protected Areas (as in Figure 3 but showing % change rather than percentage point).** a - Counterfactual summaries for the average treatment effect for the treated (ATT) direction for each plot in the Americas. Panels c and e show the same for Africa and Asia Pacific. b – country level counterfactual ATT estimates for the effect of protection summarised to the absolute change (percentage points) for the Americas (points are means and error bars 95% confidence intervals). Panels d and f show the same for Africa and Asia Pacific. In all panels colour denotes a decreased burnt area (blue), uncertain effect (grey) or increased burnt area due to protection (red).

**Figure S14. Counterfactual contrasts across the protected area network.** Counterfactual effect of protection across countries assessed in Latin America (a), Africa (b) and Asia Pacific (c). Countries in white were not included.

**Figure S15. Contrasted burnt area through time for the average Protected and Non-Protected plot.** a – In Latin America, b – in Africa and c – in Asia. Solid lines represent the predicted mean and the shaded ribbons the 95% confidence interval.

**Table S1. National level estimates of stand-replacing wildfires in tropical forests in Protected Areas.** Shown are the total burned area inside PAs, upper and lower estimates, total PA forest area and proportion of PA forest burned between 2001 and 2024. Where annual trends were significant (Mann-Kendall, p > 0.05), the trend size is displayed. Countries are labelled as per their ISO3 country codes.

| **Country** | **Region** | **Burned Area (Ha)** | **Burned Area Upper (Ha)** | **Burned Area Lower (Ha)** | **Total Forest Area (Ha)** | **Proportion Burned (%)** | **Annual Trend Size (Ha/Yr)** |
| --- | --- | --- | --- | --- | --- | --- | --- |
| AGO | AFR | 74 | NA | NA | 167655 | 0.04 | 0.5 |
| ARG | LAM | 2789 | 2369 | 3143 | 626798 | 0.44 | 4.2 |
| AUS | SEA-AUS | 5509 | 4626 | 6130 | 1197747 | 0.46 | 6.1 |
| BDI | AFR | 2 | NA | NA | 56351 | 0.00 | NA |
| BGD | SEA-AUS | 55 | 28 | 108 | 87021 | 0.06 | NA |
| BLZ | LAM | 17688 | 15919 | 19227 | 648102 | 2.73 | NA |
| BOL | LAM | 389017 | 357631 | 412816 | 9085636 | 4.28 | 485.5 |
| BRA | LAM | 2655976 | 2508817 | 2775424 | 203323075 | 1.31 | 4488.7 |
| BRN | SEA-AUS | 75 | 44 | 98 | 116268 | 0.06 | NA |
| BTN | SEA-AUS | 19 | 13 | 28 | 97486 | 0.02 | NA |
| CAF | AFR | 737 | NA | NA | 543945 | 0.14 | 2.1 |
| CHN | SEA-AUS | 3489 | 1147 | 4499 | 2149226 | 0.16 | NA |
| CIV | AFR | 1680 | NA | NA | 3436373 | 0.05 | NA |
| CMR | AFR | 89 | NA | NA | 2284184 | 0.00 | 0.3 |
| COD | AFR | 71316 | NA | NA | 19034064 | 0.37 | 188.8 |
| COG | AFR | 37975 | NA | NA | 10083285 | 0.38 | 52.3 |
| COL | LAM | 34329 | 29018 | 39459 | 15614230 | 0.22 | 54.3 |
| COM | AFR | 32 | NA | NA | 32159 | 0.10 | NA |
| CRI | LAM | 2886 | 2437 | 3337 | 1053941 | 0.27 | 3.4 |
| CUB | LAM | 6967 | 5333 | 8370 | 337265 | 2.07 | 4.7 |
| DMA | LAM | 411 | 290 | 582 | 15742 | 2.61 | NA |
| DOM | LAM | 4738 | 3842 | 5578 | 285924 | 1.66 | 6.9 |
| ECU | LAM | 3003 | 2349 | 3650 | 3866783 | 0.08 | 4.8 |
| ETH | AFR | 5 | NA | NA | 487738 | 0.00 | NA |
| FJI | SEA-AUS | 1 | 1 | 2 | 27209 | 0.00 | NA |
| GAB | AFR | 683 | NA | NA | 4716123 | 0.01 | NA |
| GHA | AFR | 4128 | NA | NA | 1579542 | 0.26 | 1.1 |
| GIN | AFR | 155 | NA | NA | 431300 | 0.04 | 0.4 |
| GNQ | AFR | 30 | NA | NA | 440726 | 0.01 | NA |
| GRD | LAM | 7 | 4 | 10 | 2828 | 0.24 | NA |
| GTM | LAM | 108130 | 96411 | 120471 | 1623439 | 6.66 | 86.6 |
| GUF | LAM | 2095 | 1438 | 2675 | 4169981 | 0.05 | 2.5 |
| GUY | LAM | 1500 | 1342 | 1638 | 1750945 | 0.09 | 2.3 |
| HND | LAM | 48050 | 40419 | 55046 | 1538450 | 3.12 | 153.4 |
| HTI | LAM | 1305 | 1069 | 1463 | 27552 | 4.73 | 0.6 |
| IDN | SEA-AUS | 283014 | 249686 | 310484 | 18000302 | 1.57 | NA |
| IND | SEA-AUS | 2 | 2 | 3 | 2410 | 0.09 | NA |
| JAM | LAM | 233 | 174 | 295 | 106540 | 0.22 | NA |
| JPN | SEA-AUS | 5 | 2 | 11 | 52042 | 0.01 | NA |
| KEN | AFR | 348 | NA | NA | 1298331 | 0.03 | NA |
| KHM | SEA-AUS | 7560 | 5365 | 9930 | 2175806 | 0.35 | -12.5 |
| KNA | LAM | 1 | 0 | 1 | 3833 | 0.02 | NA |
| LAO | SEA-AUS | 958 | 499 | 1580 | 3280795 | 0.03 | 2.8 |
| LBR | AFR | 51 | NA | NA | 266371 | 0.02 | 0.1 |
| LCA | LAM | 3 | 2 | 4 | 8935 | 0.03 | NA |
| LKA | SEA-AUS | 30 | 18 | 46 | 160566 | 0.02 | 0.1 |
| MDG | AFR | 11052 | NA | NA | 3310508 | 0.33 | 49.6 |
| MEX | LAM | 43442 | 38590 | 47447 | 3534284 | 1.23 | 106.1 |
| MMR | SEA-AUS | 429 | 229 | 740 | 3005996 | 0.01 | 1.0 |
| MOZ | AFR | 2484 | NA | NA | 917490 | 0.27 | 7.4 |
| MTQ | LAM | 7 | 3 | 12 | 35572 | 0.02 | NA |
| MYS | SEA-AUS | 1788 | 1263 | 2417 | 3878304 | 0.05 | 1.4 |
| MYT | AFR | 1 | 527 | NA | 3230 | 0.04 | NA |
| NCL | SEA-AUS | 634 | NA | 709 | 83218 | 0.76 | 0.2 |
| NGA | AFR | 954 | 49265 | NA | 1740966 | 0.05 | 1.5 |
| NIC | LAM | 57603 | 57 | 66669 | 1723506 | 3.34 | 76.4 |
| NPL | SEA-AUS | 82 | 5157 | 114 | 239315 | 0.03 | -0.2 |
| PAN | LAM | 6155 | 15509 | 7259 | 1920044 | 0.32 | 12.6 |
| PER | LAM | 18881 | 2197 | 22027 | 19883257 | 0.09 | 47.7 |
| PHL | SEA-AUS | 4795 | 0 | 8207 | 2835814 | 0.17 | 9.9 |
| PLW | SEA-AUS | 0 | 606 | 1 | 3105 | 0.01 | NA |
| PNG | SEA-AUS | 943 | 599 | 1287 | 978892 | 0.10 | 0.5 |
| PRI | LAM | 765 | 31432 | 958 | 32496 | 2.35 | NA |
| PRY | LAM | 35182 | NA | 37777 | 279536 | 12.59 | 18.1 |
| RWA | AFR | 2 | 0 | NA | 114157 | 0.00 | NA |
| SGP | SEA-AUS | 1 | 0 | 1 | 2566 | 0.03 | NA |
| SLB | SEA-AUS | 0 | NA | 0 | 7105 | 0.01 | NA |
| SLE | AFR | 1136 | 0 | NA | 477806 | 0.24 | 4.8 |
| SLV | LAM | 0 | NA | 1 | 2557 | 0.02 | NA |
| SSD | AFR | 2 | 637 | NA | 118781 | 0.00 | NA |
| SUR | LAM | 754 | NA | 864 | 1340444 | 0.06 | 2.1 |
| SWZ | AFR | 0 | NA | NA | 10259 | 0.00 | NA |
| TGO | AFR | 2 | 864 | NA | 43238 | 0.00 | NA |
| THA | SEA-AUS | 1264 | 137 | 1973 | 6393268 | 0.02 | NA |
| TTO | LAM | 60 | 325 | 89 | 128252 | 0.05 | -0.1 |
| TWN | SEA-AUS | 536 | NA | 938 | 640062 | 0.08 | NA |
| TZA | AFR | 1214 | NA | NA | 1205673 | 0.10 | NA |
| UGA | AFR | 63 | 782 | NA | 653691 | 0.01 | -0.1 |
| USA | LAM | 864 | 1 | 920 | 76357 | 1.13 | NA |
| VCT | LAM | 2 | 158467 | 2 | 6336 | 0.03 | NA |
| VEN | LAM | 174928 | 350 | 189475 | 33021613 | 0.53 | 123.8 |
| VNM | SEA-AUS | 508 | 2 | 860 | 1519339 | 0.03 | NA |
| VUT | SEA-AUS | 9 | NA | 16 | 41227 | 0.02 | NA |
| ZAF | AFR | 1211 | NA | NA | 135715 | 0.89 | NA |
| ZWE | AFR | 194 | NA | NA | 19650 | 0.99 | 0.5 |

**Table S2. Impact of removing 2024 from trend analysis.** Shown are the annual trend significance (Mann-Kendall test) and slope value (Sen’s slope) for each region including and excluding the year 2024.

| **Region** | **Mann-Kendall P value (2001-2024)** | **Mann-Kendall P value (2001-2023)** | **Sen’s Slope value (2001-2024)** | **Sen’s Slope value (2001-2023)** |
| --- | --- | --- | --- | --- |
| Americas | <0.001 | <0.001 | 5147 | 4451 |
| Africa | <0.001 | <0.001 | 54 | 48 |
| Asia-Pacific | 0.31 | 0.59 | NA | NA |

**Table S3.** ISO-codes and corresponding country names used in this study.

| **ISO Code** | **Country** | **Region** |
| --- | --- | --- |
| AGO | Angola | Africa |
| ARG | Argentina | Americas |
| AUS | Australia | Asia-Pacific |
| BDI | Burundi | Africa |
| BGD | Bangladesh | Asia-Pacific |
| BLZ | Belize | Americas |
| BOL | Bolivia | Americas |
| BRA | Brazil | Americas |
| BRN | Brunei | Asia-Pacific |
| BTN | Bhutan | Asia-Pacific |
| CAF | Central African Republic | Africa |
| CHN | China | Asia-Pacific |
| CIV | Ivory Coast | Africa |
| CMR | Cameroon | Africa |
| COD | Democratic Republic of the Congo | Africa |
| COG | Republic of the Congo | Africa |
| COL | Colombia | Americas |
| COM | Comoros | Africa |
| CRI | Costa Rica | Americas |
| CUB | Cuba | Americas |
| DMA | Dominica | Americas |
| DOM | Dominican Republic | Americas |
| ECU | Ecuador | Americas |
| ETH | Ethiopia | Africa |
| FJI | Fiji | Asia-Pacific |
| GAB | Gabon | Africa |
| GHA | Ghana | Africa |
| GIN | Guinea | Africa |
| GNQ | Equatorial Guinea | Africa |
| GRD | Grenada | Americas |
| GTM | Guatemala | Americas |
| GUF | French Guiana | Americas |
| GUY | Guyana | Americas |
| HND | Honduras | Americas |
| HTI | Haiti | Americas |
| IDN | Indonesia | Asia-Pacific |
| IND | India | Asia-Pacific |
| JAM | Jamaica | Americas |
| JPN | Japan | Asia-Pacific |
| KEN | Kenya | Africa |
| KHM | Cambodia | Asia-Pacific |
| KNA | Saint Kitts and Nevis | Americas |
| LAO | Laos | Asia-Pacific |
| LBR | Liberia | Africa |
| LCA | Saint Lucia | Americas |
| LKA | Sri Lanka | Asia-Pacific |
| MDG | Madagascar | Africa |
| MEX | Mexico | Americas |
| MMR | Myanmar | Asia-Pacific |
| MOZ | Mozambique | Africa |
| MTQ | Martinique | Americas |
| MYS | Malaysia | Asia-Pacific |
| NCL | New Caledonia | Asia-Pacific |
| NGA | Nigeria | Africa |
| NIC | Nicaragua | Americas |
| NPL | Nepal | Asia-Pacific |
| PAN | Panama | Americas |
| PER | Peru | Americas |
| PHL | Philippines | Asia-Pacific |
| PLW | Palau | Asia-Pacific |
| PNG | Papua New Guinea | Asia-Pacific |
| PRI | Puerto Rico | Americas |
| PRY | Paraguay | Americas |
| RWA | Rwanda | Africa |
| SGP | Singapore | Asia-Pacific |
| SLB | Solomon Islands | Asia-Pacific |
| SLE | Sierra Leone | Africa |
| SLV | El Salvador | Americas |
| SSD | South Sudan | Africa |
| SUR | Suriname | Americas |
| SWZ | Eswatini | Africa |
| TGO | Togo | Africa |
| THA | Thailand | Asia-Pacific |
| TTO | Trinidad and Tobago | Americas |
| TWN | Taiwan | Asia-Pacific |
| TZA | United Republic of Tanzania | Africa |
| UGA | Uganda | Africa |
| USA | United States of America | Americas |
| VCT | Saint Vincent and the Grenadines | Americas |
| VEN | Venezuela | Americas |
| VNM | Vietnam | Asia-Pacific |
| VUT | Vanuatu | Asia-Pacific |
| ZAF | South Africa | Africa |
| ZWE | Zimbabwe | Africa |
